# A method to create real-like point clouds for 3D object classification

**DOI:** 10.3389/frobt.2022.1077895

**Published:** 2023-01-06

**Authors:** Vladimir Ivanovich Syryamkin, Majdi Msallam, Semen Aleksandrovich Klestov

**Affiliations:** The International Laboratory of Vision Systems, Faculty of Innovative Technologies, Department of Quality Control, National Research Tomsk State University, Tomsk, Russia

**Keywords:** robots, vision system, 3D object classification, point clouds, data augmentation, hidden surface removal

## Abstract

There are a large number of publicly available datasets of 3D data, they generally suffer from some drawbacks, such as small number of data samples, and class imbalance. Data augmentation is a set of techniques that aim to increase the size of datasets and solve such defects, and hence to overcome the problem of overfitting when training a classifier. In this paper, we propose a method to create new synthesized data by converting complete meshes into occluded 3D point clouds similar to those in real-world datasets. The proposed method involves two main steps, the first one is hidden surface removal (HSR), where the occluded parts of objects surfaces from the viewpoint of a camera are deleted. A low-complexity method has been proposed to implement HSR based on occupancy grids. The second step is a random sampling of the detected visible surfaces. The proposed two-step method is applied to a subset of ModelNet40 dataset to create a new dataset, which is then used to train and test three different deep-learning classifiers (VoxNet, PointNet, and 3DmFV). We studied classifiers performance as a function of the camera elevation angle. We also conducted another experiment to show how the newly generated data samples can improve the classification performance when they are combined with the original data during training process. Simulation results show that the proposed method enables us to create a large number of new data samples with a small size needed for storage. Results also show that the performance of classifiers is highly dependent on the elevation angle of the camera. In addition, there may exist some angles where performance degrades significantly. Furthermore, data augmentation using our created data improves the performance of classifiers not only when they are tested on the original data, but also on real data.

## 1 Introduction

A system that can handle 3D data has a wide range of applications, such as autonomous navigation (Kriegman et al., 1989; DeSouza and Kak, 2002), autonomous household robots (Sziebig et al., 2008), obstacles detection (Manduchi et al., 2005; Mane and Vhanale, 2016; Zhu et al., 2016), rescue operations (Shang and Ma, 2015; Zhai et al., 2020), inspection of chemical and radioactive contamination (Msallam and Syryamkin, 2021a), and digital X-ray tomography for PCB diagnostics (Syryamkin et al., 2020). There are several methods to represent 3D data, such as RGB-D images, meshes, point clouds, voxels, etc. Point cloud representation is one of the most preferred methods, it is widely used in a large number of applications in many areas, such as computer vision, autonomous driving, robotics (Guo et al., 2020), chemistry (DeFever et al., 2019), fluid dynamics, digital rock physics, membrane systems, medicine and other fields (Kashefi et al., 2021; Kashefi and Mukerji, 2021; Kashefi and Mukerji, 2022). A system with point clouds at its input can perform many tasks, such as 3D classification (Mohammadi et al., 2021), scene and object segmentation (Thomas et al., 2019), detection and tracking of 3D objects (Yang et al., 2022), 3D map construction (Choy et al., 2020), reconstruction of 3D objects (Liu et al., 2019; Boulch and Marlet, 2022), and prediction (Kashefi et al., 2021; Kashefi and Mukerji, 2021; Kashefi and Mukerji, 2022).

There are many publicly available datasets that contain 3D data, such as ModelNet (Wu et al., 2015), ShapeNet (Chang et al., 2015), Sydney Urban Objects dataset (De Deuge et al., 2013), McGill 3D Shape Benchmark (Siddiqi et al., 2008), etc. A good survey of existing datasets for different deep learning tasks using 3D point clouds can be found in Guo et al. (2020). Such datasets can be divided into two main categories, real-world datasets and synthesized datasets. They generally suffer from some drawbacks, such as small number of samples per class, small number of classes, and imbalance between classes in terms of the number of samples. Objects in synthesized datasets are complete without any occlusion, they are also separated from any background. On the other hand, shapes in real-world datasets may be accompanied by background noise, and they are usually incomplete, i.e. only part of their surface visible from the camera’s viewpoint is available.

Synthesized data is a useful technology that allows to speed up research and reduce its cost, it is currently getting accelerating interest in a large number of applications, especially in machine learning systems, and medical applications where data is usually private, and obtaining a large amount of real data is a laborious task. However, more research is still needed to fully understand the opportunities and limitations offered by synthesized data. It should also be noted that the synthesized data is not a substitute for the real data. Results from synthesized data can give a good idea about the results of real data, but in the end, the final tools that will work in real applications must be evaluated and precisely tuned using real data (Heyburn et al., 2018; Jordon et al., 2022). We believe that one solution to this issue is to make the synthesized data as similar as possible to the real one.

In this paper, we try to fill the gap between synthesized and real-world datasets. We propose a method to convert complete synthesized objects represented as meshes into real-like point clouds. The resulting shapes are synthesized in nature, but they are occluded in the same way as in the real-world datasets, where the hidden parts of objects surfaces from the viewpoint of a camera are deleted. So the main idea is to propose a camera located at some point around the object in 3D space, then to delete the hidden surfaces that are not seen from the camera’s viewpoint. To make the resulting shapes more realistic, we sample the detected visible surfaces randomly, where a number of points are selected from each elementary surface at random locations with uniform distribution. These selected points form together the output point cloud. Choosing different locations of the camera and different variations of the randomly sampled points makes it possible to create a large number of new data samples from each input object. In our opinion, the proposed method enables us to create new 3D data that can be used to solve the following problems:

1) Data augmentation: the proposed method can be applied to existing datasets of 3D data with mesh representation, and new objects can be created to overcome the problem of class imbalance and to increase the number of samples, thus improving the generalization ability of classifiers and their performance.
2) Improving the training of classifiers: the created data allows us to explore whether it is better to train a classifier using complete shapes, or occluded shapes similar to those it encounters in real applications, or a combination of both complete and occluded shapes.
3) Testing already trained classifiers: even if the newly created data does not provide any improvement to the performance of a classifier, it can be used to test already trained classifiers to predict their performance in real applications.

In order to show the benefits of the proposed method, two experiments are carried out. In the first experiment, we selected 200 objects of ModelNet40 dataset and created 2e5 new data samples, then we used the created dataset to train and test three different deep-learning classifiers, they are VoxNet (Maturana and Scherer, 2015), PointNet (Qi et al., 2017a), and 3DmFV (Ben-Shabat et al., 2018). We studied their performance with respect to the elevation angle of the camera. The three classifiers showed similar behavior, as their performance varies greatly at different elevation angles. In addition, there are dead angles at which the performance drops significantly (0° in our created dataset). To the best of our knowledge, no other dataset can provide such result. The second experiment is carried out to show how the newly generated dataset can improve classification performance when it is used to augment other datasets. A subset of 600 samples of 8 classes are selected from ModelNet40 dataset, and 15,000 new data samples are generated using the proposed method, then VoxNet and 3DmFV classifiers are trained using different variations of the original and generated samples. The trained classifiers are then tested on both original samples and generated samples, and even on real data from Sydney Urban Objects dataset. The results of the conducted experiment show that the generated data improves the performance significantly in all three tests even though other data augmentation methods were used during the training process, such as rotation, scaling and jittering. In addition, the results show that training a classifier using only complete shapes gives a poor performance when it is tested on occluded shapes. So, it is better to train a classifier using a combination of complete and occluded shapes.

The paper is organized as follows: we present related work in Section 2. In Section 3 we firstly provide a reminder of some mathematical tools that we need to describe the proposed method, then we provide its details. Simulation results and discussions are presented in Section 4, and finally, we conclude in Section 5.

## 2 Related work and comparison

### 2.1 Data augmentation

Data augmentation refers to a set of techniques that aim to improve the quality of training classifiers by increasing the size of datasets, where new data samples are generated from existing ones by making some minor perturbations to them, and this in turn allows to overcome the problem of overfitting, in which a classifier is well trained to recognize its training data but it badly recognizes new data, in other words, it has a poor generalization ability. In the literature, many methods have been proposed to augment image datasets, these methods can be divided into: geometric transformations, color space augmentations, kernel filters, mixing images, random erasing, feature space augmentation, adversarial training, generative adversarial networks (GANs), neural style transfer, and meta-learning (Shorten and Khoshgoftaar, 2019).

To augment datasets of 3D point clouds, some perturbations are usually performed on them using geometric transformations, such as rotation, flipping, scaling and translation, in addition to jittering and points dropout (Maturana and Scherer, 2015; Ben-Shabat et al., 2018; Yan et al., 2020). In (Qi et al., 2017a; Qi et al., 2017b), in addition to the previous methods, points are taken randomly with a uniform distribution from the surfaces of objects represented as meshes, where the number of selected points from an elementary surface is proportional to its area. Recently, some other methods that depend mainly on mixing point clouds have been used. Although such methods provide an improvement in performance, the resulting shapes do not resemble real shapes, and the reason behind their success is still not clear (Zhang et al., 2021). Other methods are based on learning, they automatically learn the strategy for creating new data. The reader can refer to Zhang et al. (2021) for a review of the recent work according to these two approaches.

The method proposed in this paper falls under the geometrical methods, where some parts of the input object that are hidden from a camera are deleted. The method also includes taking random samples from the surface of objects with a uniform distribution as in (Qi et al., 2017a; Qi et al., 2017b). In contrast to the methods that depend on mixing point clouds, the proposed method gives shapes that are closer to the real shapes that are captured in real applications.

### 2.2 Hidden surface removal

Determination of the visible parts of an object from a viewpoint is an old problem in computer graphics, it dates back to the early 1970s, it has many applications, such as in renderers and games. Currently, there are a very large number of methods that can be used to solve this problem, i.e. visible-surface determination, or equivalently, hidden surface removal (HSR) (Cohen-Or et al., 2003). Methods for HSR are generally divided into two classes (Hughes et al., 2014): 1) object space algorithms, which are based on comparing objects in the scene in order to arrange them and determine the hidden parts in each of them. The painter’s algorithm and binary space partitioning (BSP) trees are two examples of such methods. 2) image space algorithms, that try to find the closest surface to the camera for each pixel in the image. The most common algorithms are ray casting, and depth buffer (also called Z-buffer). Exact HSR methods have usually a high computational complexity, so several methods with lower complexity have been proposed that delete many hidden surfaces while keeping all visible ones in the so-called conservative visibility, where ‘culling’ is the most common approach, it has different types: backface culling, view-frustum culling, occlusion culling, portal culling, and detail culling. Such methods are usually used as a first step before applying an exact HSR method (Hughes et al., 2014). Recently, many methods have been proposed to improve the efficiency of culling algorithms using temporal and spatial coherence between adjacent surfaces (Kim and Lee, 2022).

Among the culling methods, the proposed method is closer to the back-face culling, where in this culling method, surfaces that are directed away from the camera are deleted. This is done by comparing the direction of the normal vector of a surface and the vector coming from the camera. However, according to this method, all surfaces that are directed towards the camera are preserved, even if they are hidden. In addition, back-face culling assumes that we know in advance the direction of normal vectors of elementary surfaces, but in the proposed method there is no such assumption. It may be useful to use the back-face culling method as a first step before applying our method to reduce complexity similarly to what is usually done when applying exact HSR methods, but some problems may arise in some cases. For example, some objects such as curtains have no “inside”, therefore the “back surfaces” of such objects may also be visible and must not be deleted.

In fact, the proposed method, as we will see in the next section, is closer to the methods of exact removal of hidden surfaces, but it differs (in its original version) in that both completely hidden and partially hidden surfaces are deleted. Here we assume that the area of an elementary surface of an object is very small compared to the total surface area, so deleting or keeping partially hidden surfaces has a small effect on the resulting shape. In the next section we will see how we can modify the proposed method to implement exact HSR, and to reduce its complexity significantly.

The proposed method cannot be considered as an object space algorithm because it deals with only one object. Actually, it has some similarities with the ray casting algorithm, which searches for intersections between the ray coming from the camera and the surface of the object under consideration, but the difference lies in the formation of such rays, where the proposed method iterates over the object points and not over the pixels of the projection plane. In addition, the proposed method is concerned only with the presence or absence of such intersections, it stops the search as soon as an intersection is found.

### 2.3 Generation of occluded 3D point clouds

In (Sun et al., 2022), ModelNet40-C dataset was generated by applying 15 types of corruptions to ModelNet40 dataset. One such corruption is occlusion, which was achieved by applying ray tracing method to samples represented as meshes, where 5 different viewpoints are chosen around objects at azimuth angles selected with equal spacing from the interval [0, 2π]. Elevation angles were randomly chosen between 30 and 60°. Occlusion is considered one of the most challenging corruptions for the majority of recognition models. So there is a great similarity between this generation method and our proposed method, but there are many differences between them, for example the method of detecting hidden surfaces is different. However, an important difference is that ModelNet40-C dataset is used in the test phase only and not in the training phase as indicated in (Sun et al., 2022), while our generated dataset is intended to improve the training of 3D point cloud recognition models, not just to evaluate their robustness against corruptions. It should also be pointed out that the idea of generating occluded samples from ModelNet40 dataset was previously proposed by us in our previous work (Msallam and Syryamkin, 2021b; Msallam and Syryamkin, 2021c) prior to the date of publication of (Sun et al., 2022).

## 3 Details of the proposed method

### 3.1 An introduction to 3D geometry

Firstly, we present a reminder of some mathematical tools that we need to describe the proposed method (Stewart et al., 2015).

A position vector $a=\langle a_{1},a_{2},a_{3}\rangle$ is a vector in 3D space whose start point is the origin of the coordinates system, it can be defined completely by its end point $A=\left(a_{1},a_{2},a_{3}\right)$ . Let a,b , and c be three position vectors defined by points A,B and C , respectively. We assume that the three points are not located on the same line in 3D space, then we can uniquely find an equation of a plane passing through the three points. The normal vector can be calculated as follows:

$$n=\langle n_{1},n_{2},n_{3}\rangle =v_{1}\times v_{2},$$ (1)

**FIGURE 1:**
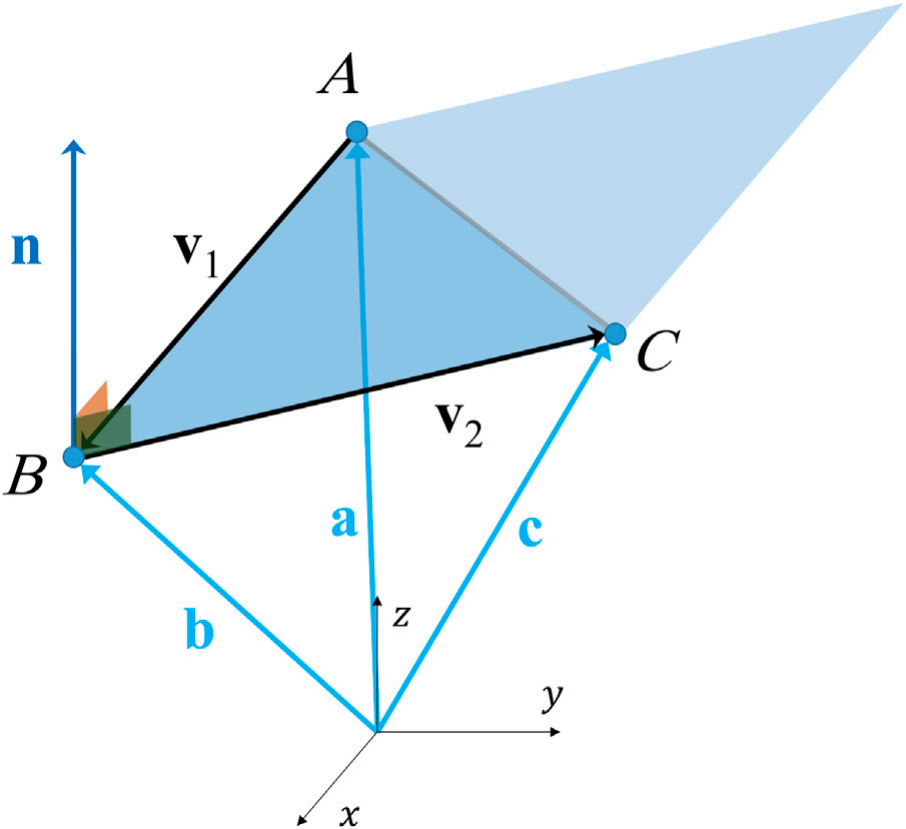
An illustration for finding the equation of a plane.

Where $v_{1}=b-a$ is the vector starting at point A and ending at point B , $v_{2}=c-b$ , and $v_{1}\times v_{2}$ is the cross product. One of the features of the cross product is that the resulting vector is perpendicular to each of the two vectors included in the product, therefore it is perpendicular to the plane formed by them as shown in Figure 1. So the projection of any position vector defined by a point from the plane onto the normal vector is constant, and the plane’s equation can be written as:

$$n_{1}x+n_{2}y+n_{3}z+d=0.$$ (2)

$\left(x,y,z\right)$ represents any point from the plane, and d is a constant that can be calculated using any point A,B or C :

d=−a.n=−b.n=−c.n, (3)

where a.n is the dot product. The area of the triangle formed by the three points A,B and C can be calculated as follows:

$$area=\frac{\left||n|\right|}{2},$$ (4)

Where ||n|| is the magnitude of the vector n . Let p and q be two different position vectors defined by the points P and Q , respectively, then any point x of the line passing through the points P and Q can be written as follows:

x=p+tu (5)

**FIGURE 2:**
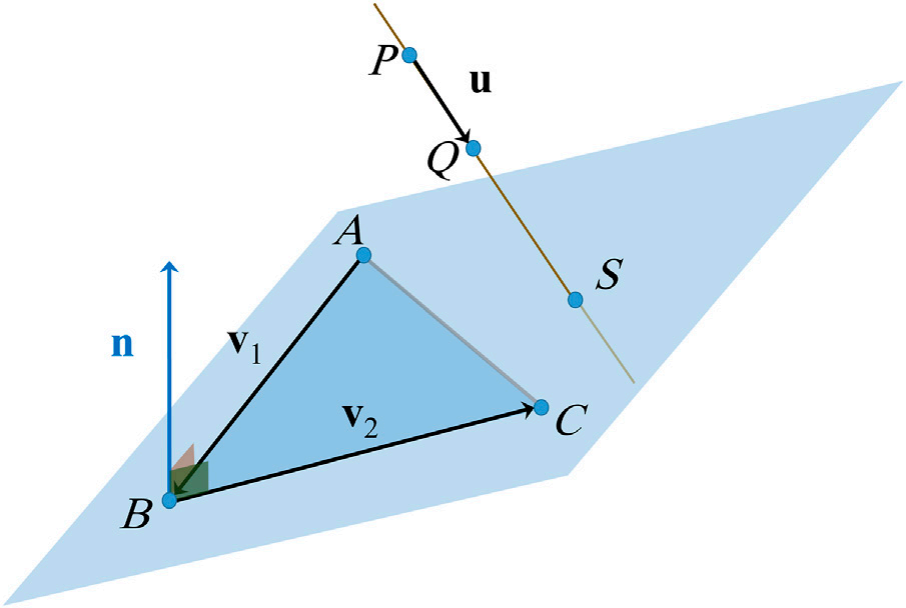
Intersection between a line and a plane formed by three points.

Where u=q−p , and t∈R is called the line’s parameter. In addition, we can find the point of intersection between a line and a plane by solving their equations jointly. By substituting Eq. 5 into Eq. 2 we get the following:

$$n.\left(p+t^{*}u\right)+d=0\Rightarrow t^{*}=-\frac{d+n.p}{n.u}.$$ (6)

provided that n.u≠0 , i.e. the line must not be parallel to the plane. Then we can substitute $t^{*}$ into the line’s equations (Eq. 5) to find the coordinates of the intersection point S as shown in Figure 2.

$$s=p+t^{*}u.$$ (7)

### 3.2 Preparation of input objects

Objects from the publicly available datasets can be used as input of the proposed method. We assume that each object is represented as a polygon mesh which is defined by two sets, the set of vertices and the set of elementary surfaces. Objects can also be created using some mathematical formulas, but a better solution is to use a CAD software such as SolidWorks which allows to create more complicated and realistic shapes.

We assume that the input objects are complete, meaning that the whole surface of each object is available. If an object is not complete, the method also works, what happens is that additional faces may be deleted during HSR step depending on the location of the camera. We also assume that each elementary surface is determined by three vertices, i.e. a triangle. Generalization to higher-order meshes is simple as each polygon can be considered as the union of many triangles.

### 3.3 Hidden surfaces removal

**FIGURE 3:**
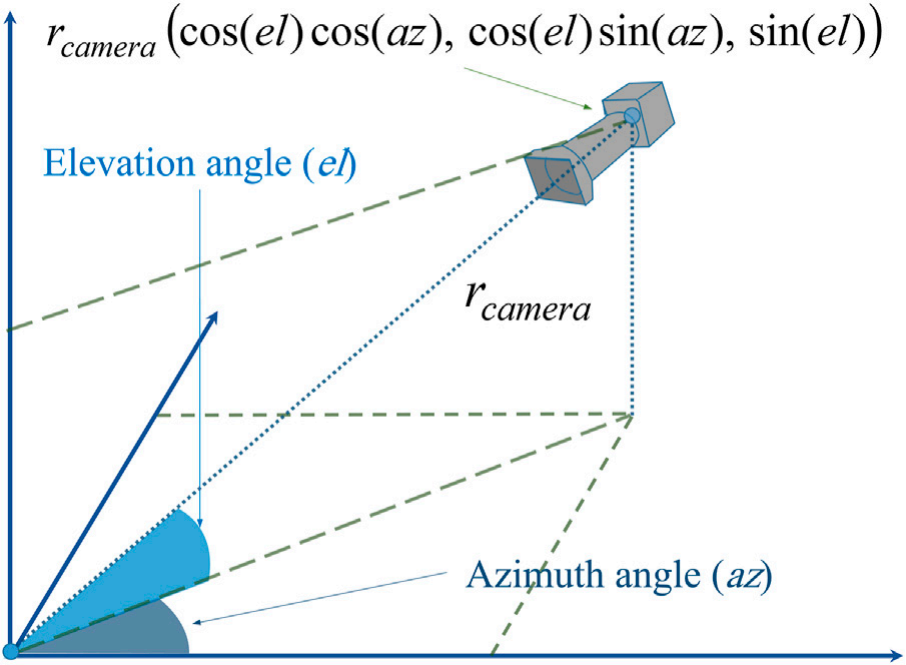
A description of the location of the camera in 3D space.

We suppose that there is a camera in the 3D space around the input object. Camera’s location is determined by three parameters: elevation angle el , azimuth angle az , and radius $r_{camera}$ as shown in Figure 3. In order to get different shapes at the output of the proposed method, different locations of the camera are selected by changing the aforementioned parameters.

An occluded vertex is a point of the object, hidden from the camera because it is located behind other parts of the object’s surface. So deleting the occluded vertices gives a result similar to capturing 3D images by the camera from the specified viewpoint. Of course, this does not completely correspond to reality, for example, the presence of a transparent surface between the camera and a point may occlude it only partially, but we will not consider such cases here. A point is considered occluded if any surface intersects the line-of-sight between it and the camera, regardless of the type of this surface.

**FIGURE 4:**
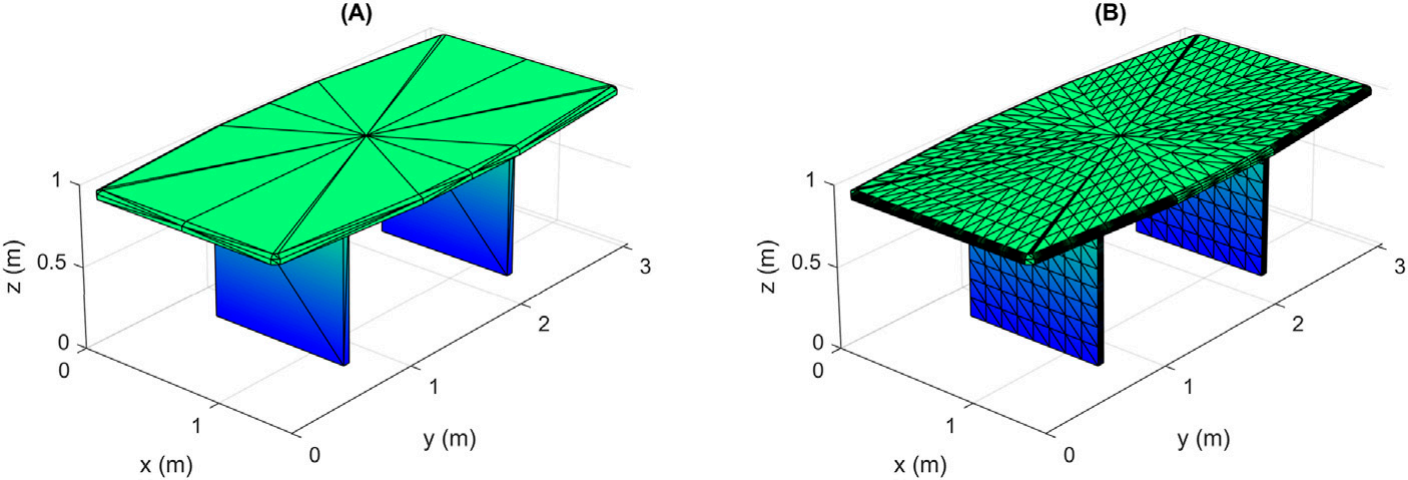
Division of elementary surfaces of large area into smaller ones: **(A)** original object, **(B)** resulting object after division.

The occluded vertices are firstly found, then they are removed with their associated elementary surfaces. We assume that the area of an elementary surface is very small compared to the whole area of the object’s surface, so deleting a surface when at least one of its vertices is occluded has a small effect on the resulting shape. If the area of an elementary surface is not relatively small, then we have to find the precise occluded part of it. One solution to this case is to further divide each large face into many smaller faces as shown in Figure 4. However, one should be careful, as this solution may increase complexity dramatically in some cases, and should be avoided if the number of the resulting points is very large. Other better solutions are presented later in Sections 3.5 and 3.6.

**FIGURE 5:**
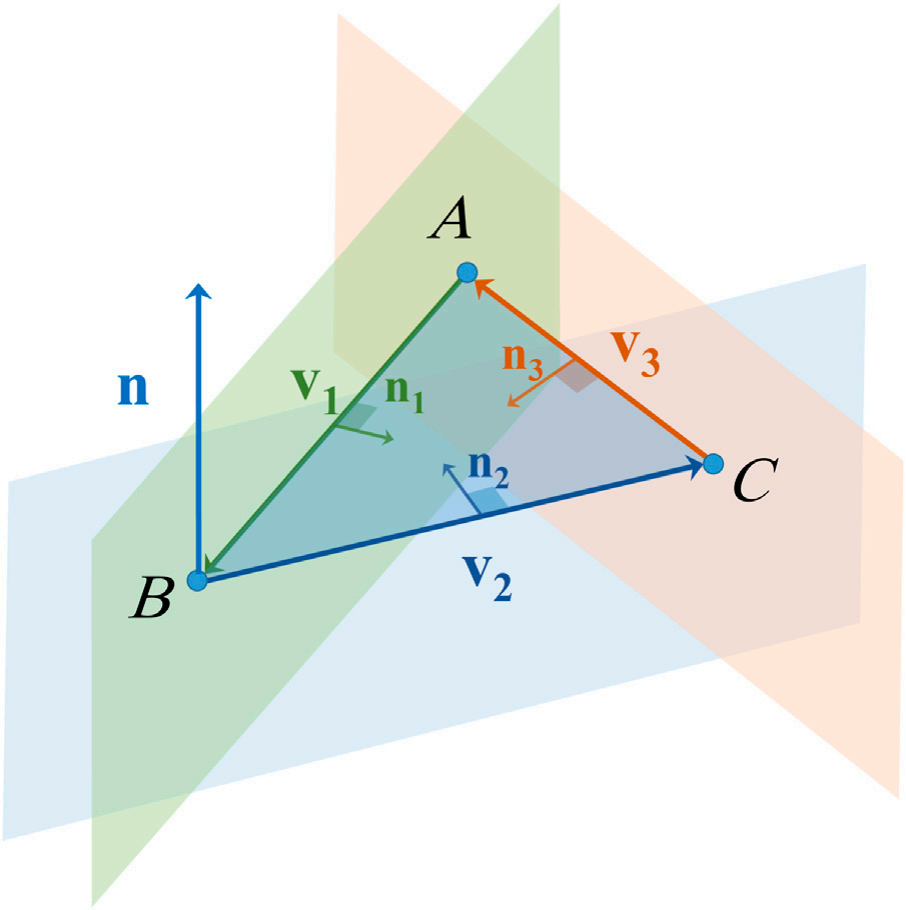
An illustration of the three perpendicular planes of an elementary surface.

To check whether a vertex is occluded or not, we form a line between this test point and the camera’s center, then we look for an intersection between the formed line and any elementary surface of the object. If such an intersection is found and the point of intersection is between the test point and the camera, then the vertex is occluded, otherwise it is visible. The equation of the plane of an elementary surface can be found using Eq. 2, the equation of the line passing through the test point and the camera can be found using Eq. 5, and the point of intersection between them can be found using Eq. 7. To check if the intersection point is located inside or outside an elementary surface, we find equations of three perpendicular planes whose normal vectors are $n_{1}$ , $n_{2}$ and $n_{3}$ as shown in Figure 5. These vectors are defined as follows:

$$n_{i}=n\times v_{i},\;i=1,2,3,$$ (8)

and the constants of the three planes can be found as follows:

$$d_{1}=-a.n_{1},d_{2}=-b.n_{2},\;d_{3}=-c.n_{3}.$$ (9)

Each one of the perpendicular planes divides the plane of the elementary surface at their intersection line into two regions, the region of positive values which is indicated by the direction of its normal vectors $n_{i}$ , and the region of negative values on the other side. Now, let s be the position vector related to the intersection point S . We calculate three values using the equations of the perpendicular planes as follows:

$$val_{i}=s.n_{i}+d_{i},\;i=1,2,3.$$ (10)

if $val_{1}\geq 0\wedge val_{2}\geq 0\wedge val_{3}\geq 0$ , then the point S is inside the elementary surface, otherwise it is located outside it (Hughes et al., 2014).

### 3.4 Random sampling of the detected visible surfaces

The detected visible elementary surfaces after applying HSR (see Figure 8B) are sampled randomly with a uniform distribution. To ensure a uniform density of sampling points over the entire surface of an object, the number of points to be sampled from an elementary surface is selected proportionally to its area (Eq. 4). If the area of a surface is too small so that the required number of samples is less than 1, let’s say 0.1 for example, then we take one sample from this surface with a probability of 0.1. This can be done by generating a random number between 0 and 1 with uniform distribution, then taking a sample only if the generated value is less than 0.1. This procedure is depicted in Algorithm 1. In this way, we avoid creating point clouds with high densities in parts containing a large number of tiny surfaces. It should be noted that the distribution of captured points in real-world point clouds is not necessarily uniform. In our future work, we may consider a variable density coefficient for each elementary surface taking into account its distance to the camera and their relative orientation.

Inputs: area of the elementary surface *A*, density coefficient s
1: Compute the exact required number of points as a real number: *n* = *A.s*.
2: Compute the integer part of *n*: *N_1_* = floor(*n*).
3: Increment *N_1_* with probability equal to the decimal part of *n*: *b* = rand
4: **if** b < *n*–*N_1_* **then**
5:  *N* = *N_1_* + 1
6: **else**
7:  *N* = *N_1_*
8: **end if**

Algorithm 1 Calculating the number of points to be samples from a surface.

**FIGURE 6:**
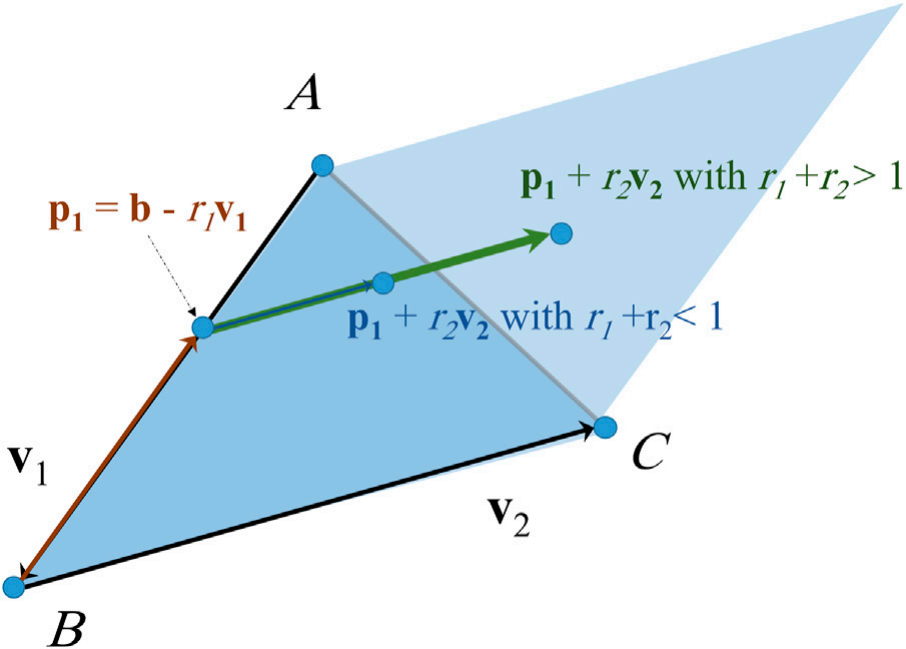
Generating a point at random location inside a triangle in 3D space.

In order to generate points at random locations inside an elementary surface we use the method described in Algorithm 2 and Figure 6, where the resulting vector $p_{2}$ is the position vector of the generated point. The condition at line 2 in Algorithm 2 ensures that all points that are generated inside the parallelogram formed by $v_{1}$ and $v_{2}$ but outside the elementary surface are moved back into the region inside it. In addition, the distribution of the generated points is uniform.

Inputs: direction vectors of the surface v1 and v2, position vector **b**
1: Generate two random numbers with uniform distribution from the interval [0, 1]: *r_1_* = rand, *r_2_* = rand
2: **if** *r_1_* + *r_2_* > **1 then**
3:  *r_1_* = 1–*r_1_*
4:  *r_2_* = 1–*r_2_*
5: **end if**
6: **p_1_** = **b**–*r_1_*. **v_1_**
7: **p_2_** = **p_1_** + *r_2_*. **v_2_**

Algorithm 2 Generating a point at a random location within a triangle in 3D space.

**FIGURE 7:**
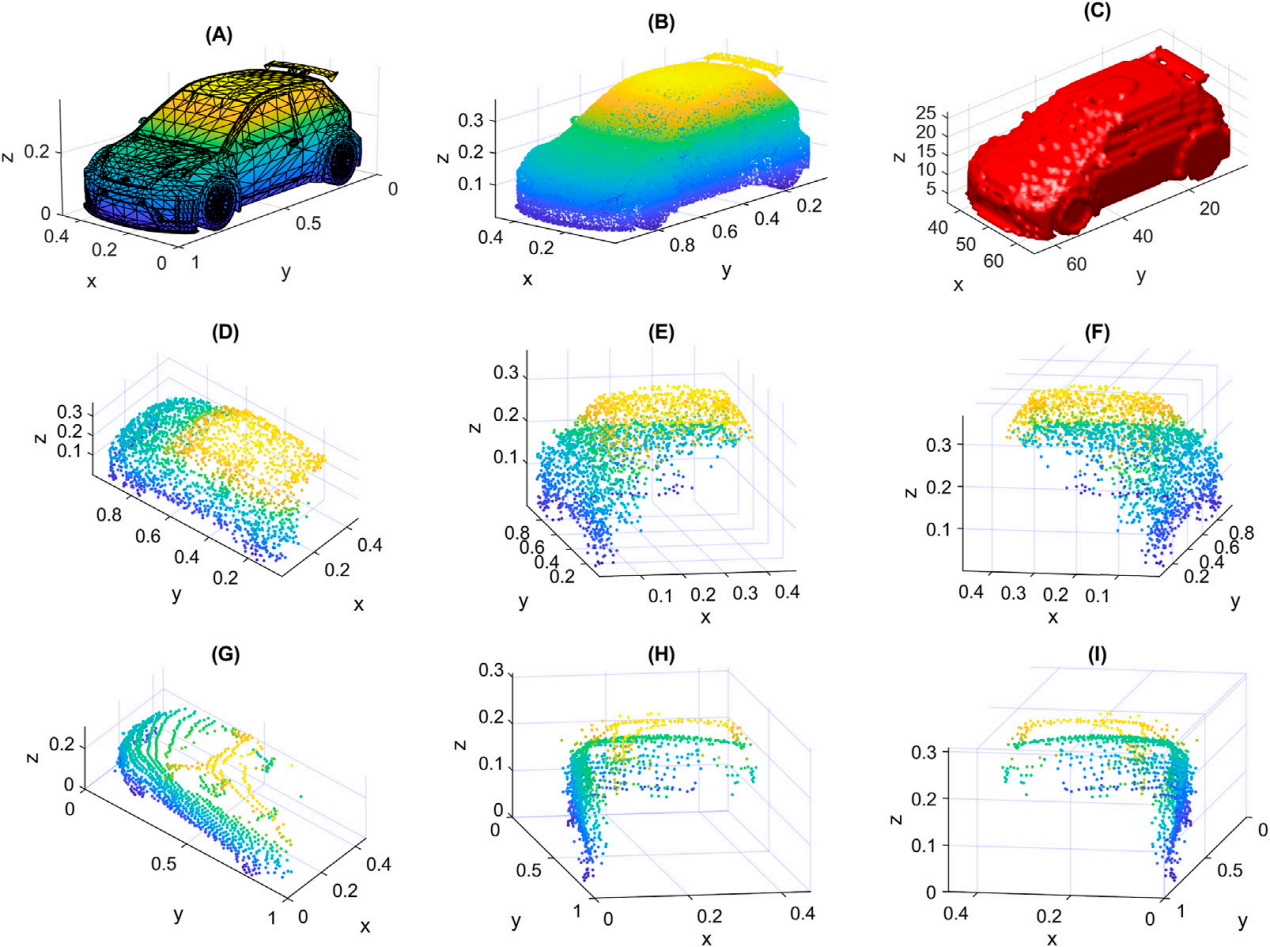
An illustrative example: **(A)** input original object from ModelNet40 dataset with mesh representation, **(B)** result of random sampling of all elementary surfaces (1e5 points), **(C)** resulted occupancy grid with size 64 × 64 × 64, **(D–F)** a created sample shown from three different viewpoints, **(G–I)** a real sample from Sydney Urban Objects dataset also shown from three different viewpoints.

**FIGURE 8:**
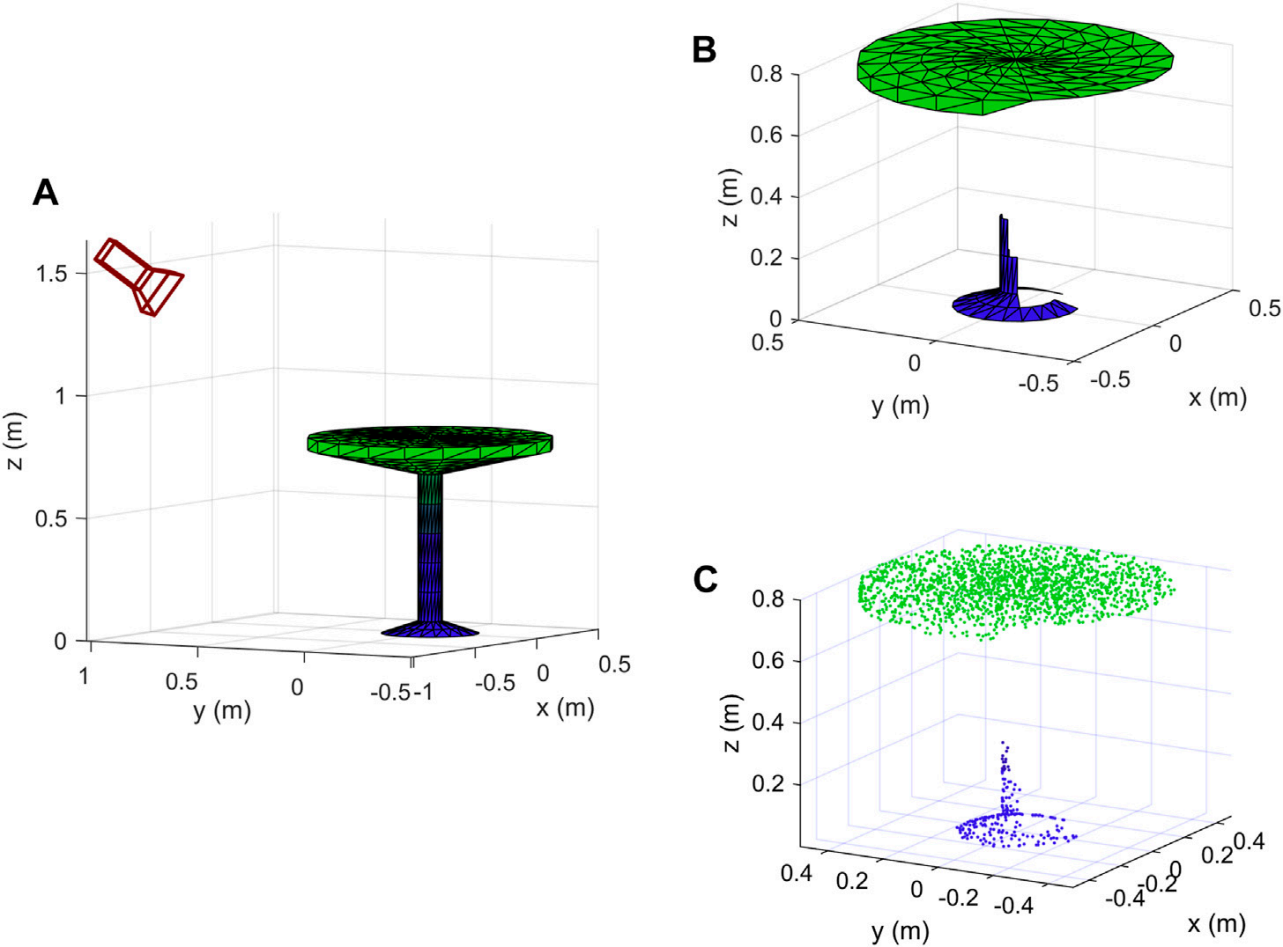
Steps of the proposed method: **(A)** original object with the camera at the specified location, **(B)** result of HSR, **(C)** resulting point cloud after random sampling of visible surfaces.

The randomly generated points form together the resulting point cloud. An example of the sampled points from all elementary surfaces of an object is shown in Figure 7B. We show in Figure 8 the results of applying the steps of the proposed method to an object. In Figure 8A we show the input object with the camera at the specified location. We also show in Figure 8B the result of HSR, and the output point cloud is shown in Figure 8C. The similarity of the newly created data samples to real data is illustrated in Figures 7D‐I, where a created sample is shown in Figure 7D‐I from three different view angles, we also show a real data sample from Sydney Urban Objects dataset of the same class “car” in Figures 7G–I from three different view angles. We note that the occlusion of the two samples is almost the same. However, the points of the real sample are distributed according to some linear patterns, which usually happens when data is captured using a LiDAR scanner. The new data can also be generated with such patterns by additional step as in (Sun et al., 2022). The samples are scaled so that their maximum dimension is 1.

The complexity of random sampling is of the order of the number of elemental surfaces $O\left(M\right)$ . Practically, this process can be performed only once for each input object, and when running the generation algorithm the process reduces to a simple selection of the points that belong to the visible surfaces detected by HSR, that is, there is no need to re-sample surfaces for each new generated object. The number of times the vectors **p1** and **p2** are calculated is equal to the number of points to be generated, which is a controlled parameter. As for calculating the equations of the elemental surfaces, this also can be performed only once as a preprocessing stage before running the generation algorithm.

### 3.5 Exact HSR

Performing HSR before the random sampling of visible surfaces has some drawbacks, especially when the object has many elementary surfaces with large areas. The proposed solution, based on the division of such surfaces into a large number of small surfaces as shown in Figure 4, can greatly increase the complexity. In addition, HSR described in Section 3.3 is not precise, since both partially and completely hidden surfaces are removed. These problems can be solved by making a modification to the proposed method, which is to change the order in which the two steps (random sampling and HSR) are performed, as follows: at first, all elementary surfaces are sampled with the required density, then HSR is applied to the generated points and not to the original vertices of the object. During HSR, the occlusion of the generated points caused by the elementary surfaces is detected, then all hidden points are deleted, and all visible points are kept to form the output point cloud.

The modification made to the method has two main advantages. Firstly, it allows exact deletion of hidden surfaces because the points are checked independently. Only the hidden points of a surface will be deleted, while the visible points of the same surface will be preserved. Secondly, this modification reduces complexity, especially when the number of original points of the object is very large. Instead of iterating over the object’s points, we iterate only over the generated points whose number is under control.

### 3.6 Complexity reduction of hidden surface removal

The proposed method of HSR (Section 3.3) is in principle based on comprehensive search, where for each vertex, all elementary surfaces are checked until an occluding one is found. So the complexity is of the order $O\left(N.M\right)$ , where N is the number of vertices, and M is the number of surfaces. The number of calculations is maximum of the from k.N.M when all vertices are visible, as in the case of a planar object with a camera outside its plane. But in the general case, the number of calculations is less than this maximum limit, as the search stops once an occluding surface is found, and there is no need to check other surfaces. However, the complexity is still high, and it is very necessary to think of some solutions to reduce it. In the following, we provide some suggestions to reduce complexity.

*The first suggestion* is to calculate the distances between the camera and the planes of elementary surfaces. This calculation can be done with $O\left(M\right)$ complexity for each location of the camera. These calculated distances are then compared with the distance between the camera and the point being tested for occlusion, and only the surfaces with lower distances are considered and the others are excluded. A surface whose all points are farther from the camera than a point cannot occlude this point.

*The second suggestion* is similar to the first one, but instead of calculating the distance between the camera and the plane of a surface, we choose a set of points from inside the surface and calculate the minimum distance between them and the camera. We assume that this minimum distance is a good approximation of the distance between the surface and the camera. Then, all surfaces whose distances are greater than the distance between the camera and the considered point are excluded. A good approximation can be guaranteed by practically selecting a sufficiently high value of the density of the generated point. However, very large densities are not recommended because the complexity of calculating distances is of the order of the number of the generated points. It should be noted that the same points that were generated to form the output point cloud can be used here to calculate the distances. It should also be noted that the set of surfaces excluded according to the first suggestion is a subset of the set of surfaces excluded according to the second suggestion, so in our simulation we implemented the second suggestion and found that it can reduce the execution time by more than 50% as shown in Table 1A.

**TABLE 1:** (A) Comparison of the execution time of the original and modified HSR, (B) Comparison of execution time between exact HSR and modified HSR based on occupancy grids for different grid sizes.

Object	#Faces	#Points	Execution time (s)		Reduction (%)	
			$t_{original}$	$t_{modofied}$		
A				
Car (5)	18,014	12,759	228.1	94.8	58.4	
Car (6)	13,164	19,475	343.2	159.0	53.7	
Chair (3)	2,628	7,884	12.5	5.6	55.2	
Lamp (3)	5,903	5,364	29.9	12.4	58.5	

Object	#Faces	#Sampling points	Execution time (s)			Reduction (%)	
			Exact HSR	OG_32	OG_64	OG_32	OG_64
B							
Car (5)	18,014	1,000	18.5	0.3	0.3	98.4	98.4
		10,000	163.8	1.3	2.4	99.2	98.5
		50,000	812.7	1.5	6.3	99.8	99.2
Car (6)	13,164	5,000	24.2	0.7	1.1	97.1	95.5
		20,000	96.5	1.3	3.7	98.7	96.2
		100,000	493.0	1.6	4.8	99.7	99.0

OG_n: HSR, using occupancy grids with size n × n × n

*The third suggestion* is a method that greatly reduces computational complexity but is less accurate, it depends on converting the object into an occupancy grid as shown in Figure 7C, then searching for the occluded cells instead of searching for the occluded points, and finally, all points belonging to an occluded cell are also considered occluded. The advantage of this method is that its complexity is limited by the number of occupied cells of the whole object, and therefore it is best suited to objects that have a very large number of points. The accuracy of HSR using occupancy grids is dependent on the dimensions of the grid. Accuracy can be improved by increasing the grid dimensions within the permissible limits of complexity. In our simulation we firstly used grids of size 32 × 32 × 32 as a good compromise between accuracy and complexity, then we used grids of size 64 × 64 × 64 which gave better results with acceptable increase in complexity.

In Table 1A we provide a comparison between the execution time of the original method $t_{original}$ and that of its modified version according to the second suggestion $t_{modified}$ . The comparison is made using objects from ModelNet40 dataset whose names are shown in the first column, where the number in parentheses indicates the object index in the dataset. In the last column, we show the amount of reduction in execution time, it is equal to $1-t_{modified}/t_{original}$ , and it is around 55% as we can see.

In Table 1B we compare the execution time of the exact HSR (Section 3.5) with its modified version according to the third suggestion (based on occupancy grids). We note from the third column that we are free to generate any required number of points for the entire object. We also note that the use of occupancy grids greatly reduces the execution time especially when the number of points is very large. This allows us to create very dense point clouds. Therefore, we choose this method to create our new data and to obtain the results shown in the next section.

## 4 Results and discussion

### 4.1 Specifications of the created data

**FIGURE 9:**
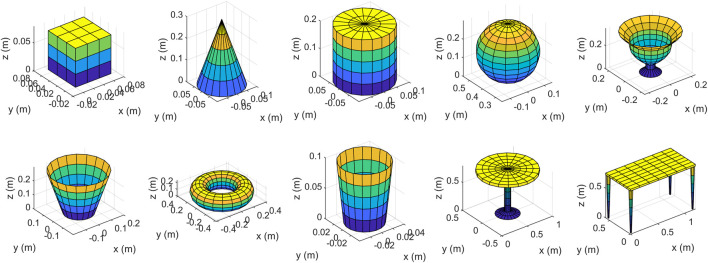
Some simple objects that we designed to test the proposed method.

We implemented the proposed method described in Section 3 using both our own created objects and data from publicly available datasets. At first, we used the simple objects shown in Figure 9 as input, we created them using mathematical formulas and plotted them in Matlab, then from each of them we created 1,000 new data samples using the proposed method. Secondly, we selected 5 objects from each of the 40 classes of ModelNet40 dataset (Wu et al., 2015), and also generated 1,000 samples from each input object. The total number of different camera locations that we selected is 100. Elevation and azimuth angles are chosen using the following formulas:

$$el_{i}=\left(i-1\right)\times 10-90,\;i=\left\{1,2,\ldots ,19\right\},$$ (11)

$$az_{ij}=\left(j-1\right)\times \frac{2\pi }{N_{i}},\;j=\left\{1,2,\ldots ,N_{i}\right\},\;N_{i}=\min\left(i,\;20-i\right).$$ (12)

The radius of the camera location is chosen so that it is located far enough from the object (4 times the object’s maximum dimension). At each camera location we generated 10 data samples for each object, they differ from each other by the locations of random points as described in Section 3.4. The size of occupancy grids for this experiment is 32 × 32 × 32.

**FIGURE 10:**
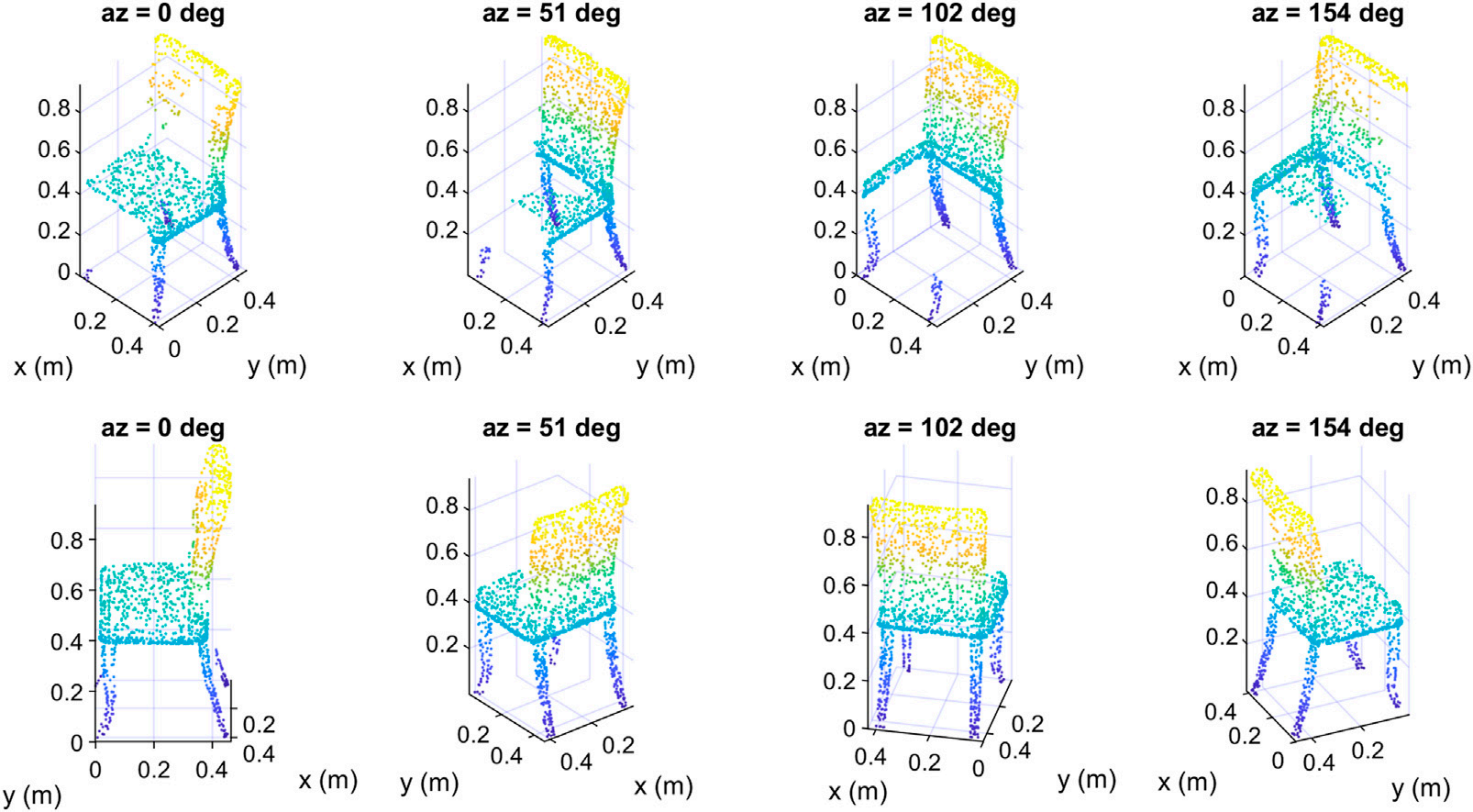
Some data samples of the class “chair” of the created dataset at different azimuth angles: top) fixed viewpoint, down) camera’s viewpoint.

**FIGURE 11:**
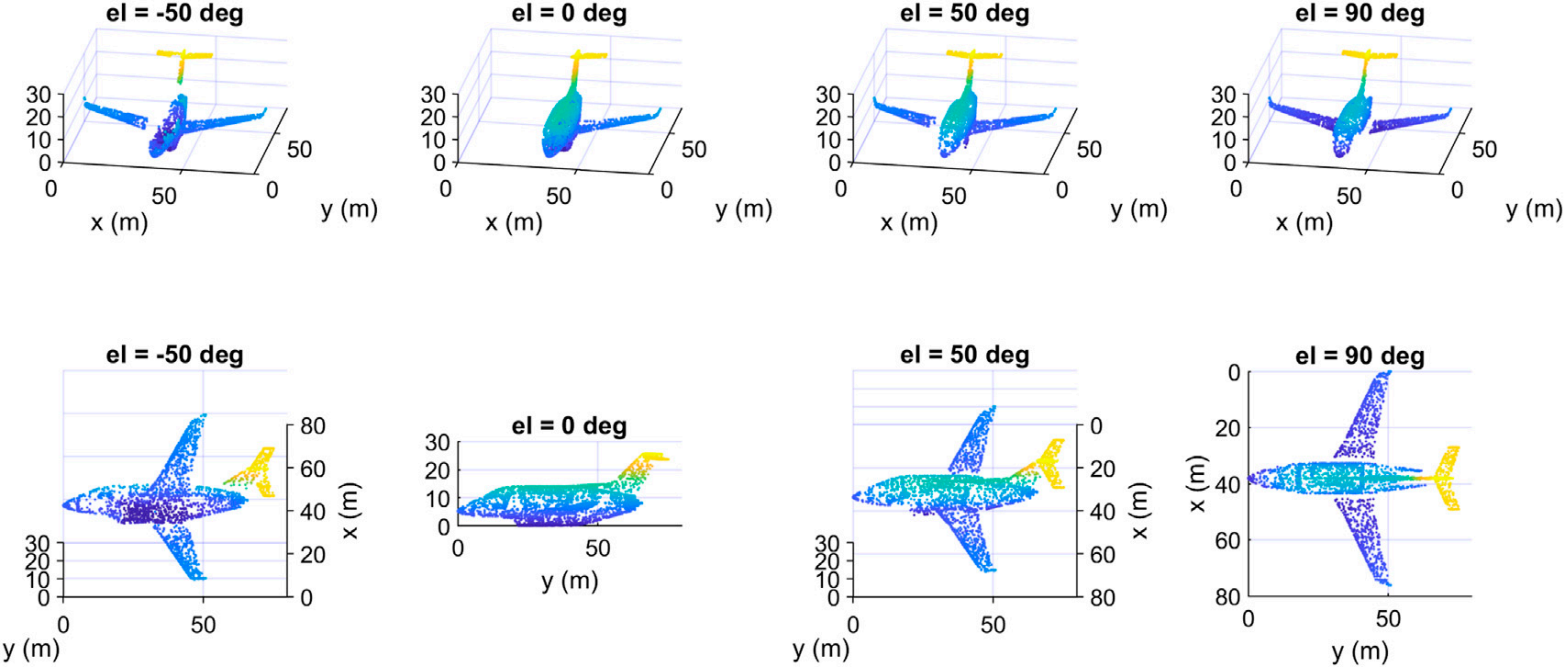
Some data samples of the class “airplane” of the created dataset at different elevation angles: top) fixed viewpoint, down) camera’s viewpoint.

We show in Figure 10 four data samples that we created from the same input object but at different azimuth angles. The upper row in Figure 10 contains the samples as seen from a fixed viewpoint. The same samples are redrawn in the second row but as seen from the camera’s viewpoint, so each column contains only one data sample shown from two different viewpoints. Although the four samples represent the same object, they are different from each other, as different parts of the input object were deleted to create them depending on the location of the camera. We note that from the camera’s viewpoint, the samples seem complete, but in fact they are incomplete, as the hidden parts of their surfaces have been deleted, which is obvious from the fixed viewpoint (upper row). In Figure 11 we show four other data samples that were created from another input object at different elevation angles. Here also, each column shows exactly the same created sample but from two different view angles.

And in Figure 11 we show some other data samples of another object at different elevation angles. The upper row in Figures 10, 11 contains the samples as seen from a fixed viewpoint, while the lower row contains the same samples but from the camera’s viewpoint.

In (Guo et al., 2020) a good comparison between a number of datasets dedicated for 3D object classification is presented. In Table 2 we provide this comparison and add information about our created dataset using the proposed method with objects from ModelNet40 dataset as input. We also add two columns, the size required to store the dataset in Megabytes (MB), and the average number of data samples per 1 MB. We notice that our created dataset has a larger number of samples compared to other synthesized datasets that have almost the same size required for storage.

**TABLE 2:** Comparison between our dataset and other datasets for 3D object classification.

Dataset name	#Samples	#Classes	Type	Representation	Size (MB)	#Samples/ 1 MB
McGill Benchmark	427	19	Synthesized	Mesh	412	1.04
Sydney Urban Objects	631	14	Real-World	Point Cloud	9.66	65.32
ModelNet10	4,899	10	Synthesized	Mesh	2,170	2.26
ModelNet40	12,311	40	Synthesized	Mesh	9,100	1.35
ShapeNet	57,454	55	Synthesized	Mesh	92,900	0.62
Our created dataset	200,000	40	Synthesized	Point Cloud	6,940	28.82

### 4.2 Classification performance vs. elevation angle of the camera

In this section, we show how the created dataset can be used to obtain some results that cannot be obtained directly using publicly available datasets. To be more precise, we will study the performance of some classifiers of 3D objects as a function of the elevation angle of the camera. Such result may be of interest in many real applications where the camera is located at almost the same elevation angle with respect to the objects in the scene. For example, in flying robots that capture images of the ground, the camera elevation angle is close to 90°, while in autonomous driving applications, the angle is close to 0°.

We use a subset of the generated dataset as described in Section 4.1 to train three different deep learning classifiers, they are VoxNet, PointNet, and 3DmFV. Another subset of the generated data is used for testing. The subsets are selected based on the elevation angles of the camera locations, where we choose data samples at elevation angles {±90, ±70, ±50, ±20} for training, and at {±80, ±60, ±40, ±30, ±10, 0} for testing. The total classification accuracy over all classes is shown in Table 3A for the three classifiers. Although the performance of the classifiers is different, the aim here is not to compare them but to show the relationship between their performance and the elevation angle.

**TABLE 3:** Overall classification accuracy of the conducted experiments: (A) performance vs. elevation angle of the camera, (B) performance improvement by data augmentation.

OA (%)	Elevation angle (degrees)										
	−80	−60	−40	−30	−10	0	10	30	40	60	80
A											
VoxNet	69.7	71.1	71.6	74.4	69.0	50.3	60.4	69.3	69.0	68.0	63.9
PointNet	65.6	64.9	74.4	76.9	75.5	60.6	75.6	78.3	78.2	74.8	68.0
3DmFV	97.6	97.3	98.0	98.6	97.6	86.8	97.7	99.1	98.5	97.0	97.3

	Training data	Test 1 (original) OA		Test 2 (generated) OA		Test 3 (real data) Acc					
		VoxNet	3DmFV	VoxNet	3DmFV	VoxNet		3DmFV		3DmFV^a^	
						car	4wd	car	4wd	car	4wd
B											
C_1_	Generated	81.0	87.0	76.3	95.2	**37.5**	**66.7**	**77.3**	**42.9**	**88.6**	66.7
C_2_	ModelNet8^b^	82.0	88.5	45.9	81.3	0	0	17.1	19.1	36.4	42.9
C_3_	ModelNet8 + generated	**87.5**	89.5	**79.7**	**95.7**	20.5	38.1	63.6	38.1	76.1	**81.0**
	ModelNet40	69.0^c^/83.0^d^	91.6^e^	**—**	**—**	**—**	**—**	**—**	**—**	**—**	**—**

OA: overall accuracy; Acc: accuracy; Ci: Classifier i.

^a^ The classifier uses 1,024 points, while in all other cases it uses 12,000 points.

^b^ ModelNet8 is the subset we selected from ModelNet40.

^c^ (Maturana and Scherer, 2015) without rotation augmentation.

^d^ (Maturana and Scherer, 2015) with rotation augmentation.

^e^ (Ben-Shabat et al., 2018) with 2048 points.

**FIGURE 12:**
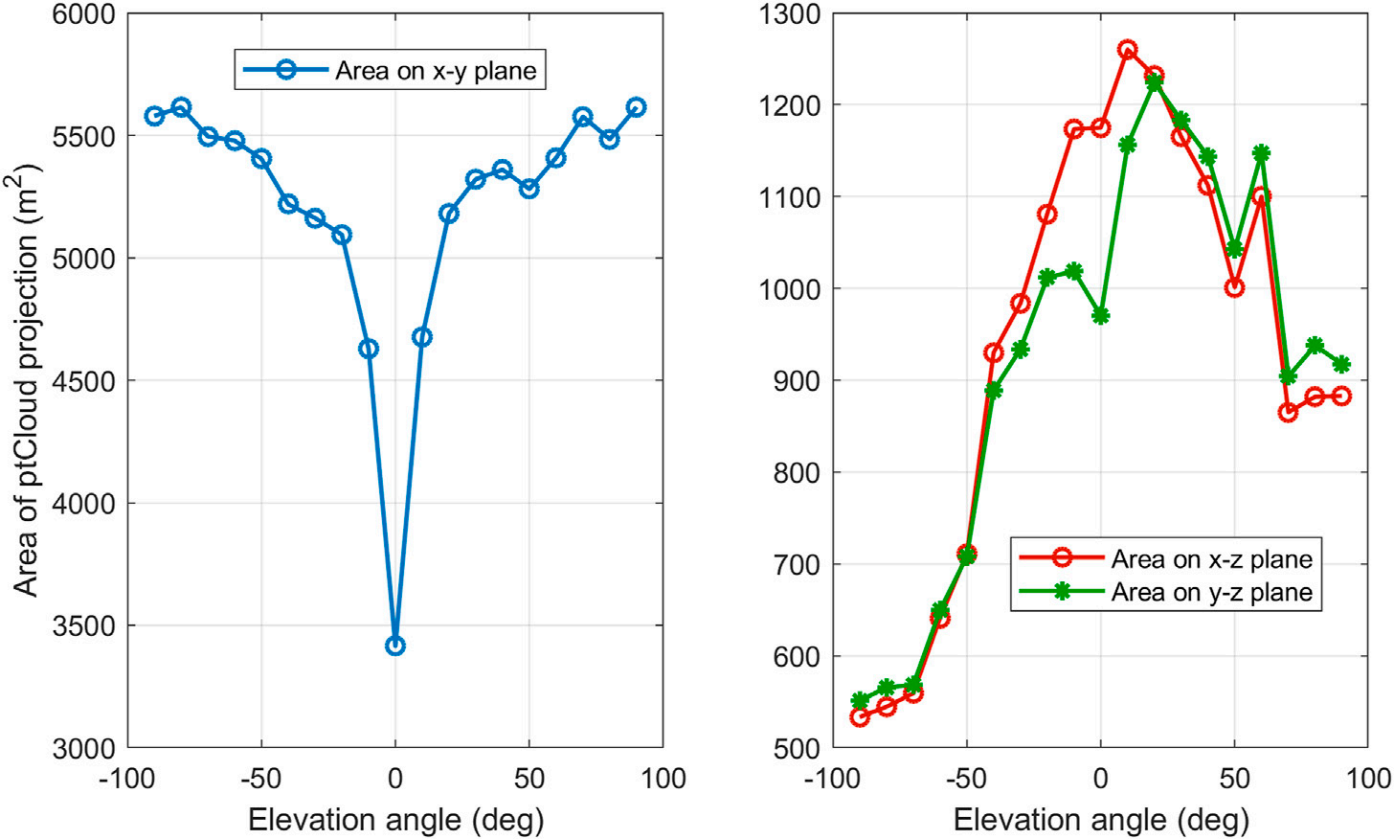
Projected area of the visible part of an object onto the three planes of the coordinates system as a function of the camera elevation angle.

From Table 3A we notice that the performance is highly dependent on the elevation angle, this means that the ability to correctly recognize an object depends on the viewpoint from which it is seen. We also note that the performance degrades significantly around elevation angle 0 for all classifiers. It also degrades at ±60 and ±80. The reason for this phenomenon, as we think, is that the area of the visible part of the object’s surface differs from one elevation angle to another. Figure 12 shows the relationship between the elevation angle and the projection area of the visible part of an ‘airplane’ onto the three coordinate planes. The curve on the left is the projection area onto the x-y plane. We found that for a large number of objects this area is relatively very small at elevation 0, which may explain the performance degradation at this angle. We also found that the area of the other two projections onto the other two planes is relatively small around the ± 90 angles, which may explain the degradation in performance around these two angles.

### 4.3 Performance improvement by data augmentation

In order to verify the importance of the generated data according to the proposed method, we conducted an experiment as follows: a subset is selected from ModelNet40 dataset as an input to the proposed generation method, where 8 classes out of 40 are chosen, they are {airplane, bed, bookshelf, bottle, car, chair, lamp, table}. The number of selected samples from each class is 75, divided into: 40 for training, 10 for validation, and 25 for testing. So the total number of selected samples is 600, represented as meshes. We call the selected subset ModelNet8 since it has only 8 classes, it is a balanced dataset with a relatively small number of samples. The number of samples was intentionally chosen so that the resulting set is relatively small and insufficient to train classifiers. In such a case, the need to augment data arises. If the number of samples was originally large, then data augmentation methods will provide a slight improvement in performance. We applied the proposed method to generate 25 new objects from each input one, where each new object corresponds to a different location of the camera. As for the elevation angles, we chose the values {±80, ±40, 0} in degrees, while for the azimuth angles, we chose the values {0, 72, 144, 216, 288} in degrees. So, we have 5 elevation angles and 5 azimuth angles, and thus the number of different camera locations is 25. The angles are chosen so that the resulting samples are as different as possible from each other, and this is done by making the camera locations farther apart. The choice of camera locations usually affects the quality of the resulting data, for instance, close locations will most likely give very similar samples. Accordingly, elevation angles were first selected with equal spacing from the interval [−π/2, π/2], then azimuth angles were chosen with equal spacing from the interval [0, 2π] at each elevation angle. For some applications, elevation angles can be selected within a specified range according to the angles at which objects are usually seen in the real world, for example in automated driving applications, camera elevation angles can be selected between 0 and 50°. The size of occupancy grids that are used for HSR in this experiment is 64 × 64 × 64.

Consequently, we get 600 original data samples from ModelNet40 dataset, in addition to 15,000 new samples generated using the proposed method. We employ these two datasets with different configurations to form three different training sets as follows:

− Training set 1: it only contains samples from the generated data (8,000 samples). The resulting trained classifier in this case is called Classifier 1.
− Training set 2: it contains samples from the original dataset (320 samples), they are converted into point clouds by sampling each of their elementary surfaces randomly with a uniform distribution. The result is called Classifier 2.
− Training set 3: it is the union of the sets 1 and 2 (8,320 samples). The result is Classifier 3.

These sets are used to train VoxNet and 3DmFV classifiers. As a result of the training process, and for each one of these two models, we get three classifiers that have the same structure but with different parameters. We then evaluate the performance of the resulting classifiers to recognize the test set of the original dataset (200 samples). The overall accuracy is shown in Table 3B in the columns below the label “Test 1 (original)”. In this table, the shaded cells indicate the results that we got without using the generated data either in the training process or in the testing process.

In all three cases, even when the classifiers were trained using the original data only, the data is augmented using some geometrical methods, namely rotation, scaling, and jittering, which improved performance to some extent, but as we can see from the results, there is still room to improve performance by augmenting the original data using our generated data. This is clear in Table 3B, where we notice an improvement in the performance of VoxNet classifier from 82.0% using Classifier 2 to 87.5% using using Classifier 3. The performance of 3DmFV classifier is also improved from 88.5% to 89.5%. The result of Classifier 1 is also so interesting, it shows that training a classifier using only occluded shapes makes it able to recognize complete shapes with a small loss in performance. However, the opposite is not necessarily true as we will see in the next section.

In Table 3B, we also compare our results with the performance of the classifiers when they are trained using the entire ModelNet40 dataset. We note that the performance of ModelNet8 dataset is slightly lower than that of ModelNet40, but it can serve as a good approximation of ModelNet40 performance. The slight performance loss is due to the reduction in the number of samples in ModelNet8.

### 4.4 Prediction of classification performance on real data

Now, we evaluate the performance of the trained classifiers, as described in the previous section, using test samples only from the generated data (5,000 samples). The objective of this test is to predict the performance of classifiers in real applications, based on the assumption that the generated samples are similar to the real data, since they are occluded in the same way. The results are shown in Table 3B in the columns below the label “Test 2 (generated)”.

We notice that Classifiers 1 and 3 performed relatively well, while there is a huge loss in performance using Classifier 2 which is trained using only original samples. We therefore conclude that a classifier that is trained using only complete shapes will have a poor performance in recognizing occluded shapes, compared to the case when it is trained using occluded shapes.

From the results of Test 2 we expect that Classifiers 1, and 3 will perform better than Classifier 2 in real applications. Of course, it is indispensable to test the classifiers on real data to verify the validity of this conclusion.

Actually, we tested the trained classifiers using data samples from Sydney Urban Objects dataset (De Deuge et al., 2013). This dataset has many classes for different types of cars, such as “4wd,” “bus,” “car,” “truck,” “ute,” and “van.” However, the classifiers we’ve trained have only one output for all types of cars, so when they are tested on Sydney Urban Objects dataset we would expect that all the samples of these classes will be labeled as instances of the class ‘car’. In this case, the true positive (TP) of the class ‘4wd’ for example will be the number of its samples that are correctly labeled as instances of the class “car.” A false positive (FP) can be defined as the number of samples of the other classes (other than the six mentioned earlier) that are wrongly labeled as instances of the class “car”.

We show the classification accuracy of the classes “car” and “4wd” in Table 3B in the columns below the label “Test 3 (real data) .” We note the following: 1) The performance in general is lower compared to the results of Test 2. We conclude that it is preferable to include some real data samples when training a classifier in addition to the synthesized samples. 2) Classifier 2 which is trained using only complete shapes was unable to recognize most of the test data. 3) Classifiers 1 and 3 that were trained using occluded shapes have indeed recognized the test samples better than Classifier 2, with a very good performance in some cases.

If we consider the aforementioned definitions of TP and FP, we can calculate average F_1_ score for the above-mentioned six classes of Sydney Urban Objects dataset that represent different types of cars. The total number of samples in these classes in 188, and the resulted average F_1_ score using our trained 3DmFV classifier with 1,024 points is 79.3%, which is higher than the average F_1_ score found in (Maturana and Scherer, 2015; Ben-Shabat et al., 2018). We emphasize that we trained the classifiers using synthesized data only, and tested them on real data.

In Test 3, the best results, which are shown in the last two columns of Table 3B, are provided by 3DmFV classifier when it is trained using 1,024 points in the training samples, while in all other cases it was trained using 12,000 points. The reason behind this improvement, as we think, is that the value 1,024 is closer to the number of points in the real test samples. So, it is preferable to select the training samples with density close to that of the shapes that will be encountered in real applications.

A general comparison between Classifiers 1 and 3 shows that in general it is preferable to train a classifier using a combination of both complete and occluded objects.

Finally, it should be noted that we do not claim that the newly created samples are a substitute for the original samples. Replacing a complete original sample with only one occluded sample is simply a waste of data and is expected to lead to worse classification performance. The generated samples are supportive of the original samples, they improve the quality of training classifiers when they are added to the original samples, as we have found that it is best to train classifiers using a combination of complete and occluded samples. Using occluded samples alone in training may cause some slight decrease in performance as seen in Table 3B, and to achieve this a sufficient number of new samples must be generated from each original sample (we generated 25 new samples from each one).

## 5 Conclusion and future work

In this paper, we provided the details of our proposed method to create synthesized 3D point clouds that have similarities to those in real-world datasets. The method consists mainly of hidden surface removal and random sampling of visible surfaces. Many modifications have been proposed to reduce its computational complexity. We implemented the proposed method firstly using our designed objects, then on data samples from the public dataset ModelNet40. Simulation results show that the proposed method enables us to create a large number of new data samples from each input object. Using only 200 objects of ModelNet40 dataset we could create 2e5 new samples to form a new dataset for 3D object classification. The created dataset is characterized by a large number of data samples per class and a small size needed for storage compared to other synthesized datasets.

We used our created dataset to train and test three different deep learning classifiers, they are VoxNet, PointNet, and 3DmFV. We evaluated their performance at different elevation angles of the camera, and found that their performance differs from one angle to another. Such result may be of interest in many real applications. Another interesting result is that the performance may degrade at some angles according to the area of the visible surface of the object from the camera’s viewpoint.

We also conducted an experiment to verify how the newly generated dataset can improve classification performance when it is used to augment other datasets. A subset of 8 classes and 600 samples are selected from ModelNet40 dataset for the experiment, and 15,000 new data samples are generated using the proposed method, then VoxNet and 3DmFV classifiers are trained using different variations of the original and generated samples. The trained classifiers are then tested on original samples and generated samples, and even on real data from Sydney Urban Objects dataset. The selected dataset, which we called ModelNet8, is a typical example of a small dataset with balanced classes. We hope that in the future this dataset will be used widely to evaluate the performance of the recognition models, as the training process can be done very quickly compared to ModelNet40 and even ModelNet10, and thus a rough estimation of the performance can be made before training the model using a large dataset which usually takes a very long time.

The results of the conducted experiment showed that the generated data improves the performance significantly in all three tests even though other data augmentation methods were used during the training process, such as rotation, scaling and jittering. In addition, the results show that training a classifier using only complete shapes will not guarantee a good performance in real applications. Rather, it is preferable to train classifiers using a combination of complete and occluded shapes. However, there is still a gap between the performance of the generated data and the real data. This is due to many reasons, for example, number of points, their distribution, occlusion caused by other objects, etc. Therefore, in our future work we will try to consider all such influences to create data that is more similar to real data.

## Data Availability

The raw data supporting the conclusion of this article will be made available by the authors, without undue reservation.
